# Epigenetic Age Acceleration and Cardiometabolic Biomarkers in Response to Weight‐Loss Dietary Interventions Among Obese Individuals: The MACRO Trial

**DOI:** 10.1111/acel.70224

**Published:** 2025-09-08

**Authors:** Minghao Kou, Xiang Li, Yoriko Heianza, Kirsten Dorans, Lydia Bazzano, Lu Qi

**Affiliations:** ^1^ Department of Epidemiology, Celia Scott Weatherhead School of Public Health and Tropical Medicine Tulane University New Orleans Louisiana USA; ^2^ Division of Endocrinology, Diabetes and Metabolism, Department of Medicine University of Illinois Chicago Chicago Illinois USA; ^3^ Department of Nutrition Harvard T.H. Chan School of Public Health Boston Massachusetts USA

**Keywords:** aging, cardiometabolic health, dietary intervention, DNA methylation

## Abstract

Epigenetic clocks have emerged as promising biomarkers of aging, but their responsiveness to lifestyle interventions and relevance for short‐term changes in cardiometabolic health remain uncertain. In this study, we examined the associations between three epigenetic aging measures (DunedinPACE, PCPhenoAge acceleration, and PCGrimAge acceleration) and a broad panel of cardiometabolic biomarkers in 144 obese participants from the MACRO trial, a 12‐month weight‐loss dietary intervention comparing low‐carbohydrate and low‐fat diets. At pre‐intervention baseline, DunedinPACE was significantly associated with several cardiometabolic biomarkers (FDR [false discovery rate] < 0.05), including insulin, homeostatic model assessment for insulin resistance (HOMA‐IR), total cholesterol, high‐density lipoprotein cholesterol, C‐reactive protein, adiponectin, and ghrelin. These associations were substantially attenuated following the intervention, with only CRP and adiponectin remaining significant. Changes in epigenetic aging measures were not significantly associated with changes in biomarkers, nor did they mediate the effects of weight loss. Our findings highlight DunedinPACE as a sensitive biomarker of cardiometabolic health in adults with obesity but raise questions about the utility of epigenetic clocks as causal targets in short‐term lifestyle interventions. While caloric restriction may attenuate some phenotypic manifestations of biological aging, short‐term changes in epigenetic aging measures may not fully reflect underlying cardiometabolic changes. These results underscore the need for caution in interpreting epigenetic aging as a modifiable intervention target.

## Graphical abstract

In a 12‐month weight‐loss trial, DunedinPACE was associated with cardiometabolic biomarkers at baseline, but not over time. No evidence supported epigenetic clocks as mediators of intervention effects, raising caution about their causal mechanisms as short‐term intervention targets for healthy aging.

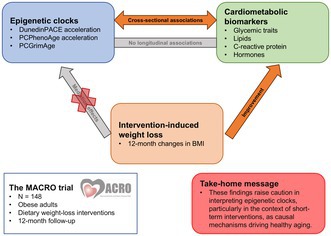

## 1 Introduction

Aging progresses at varying rates both within and between individuals (Elliott et al. 2021; Tian et al. 2023). Epigenetic aging clocks have been developed based on a well‐characterized hallmark of aging, DNA methylation, as measures of biological aging (López‐Otín et al. 2023; Horvath and Raj 2018; Fransquet et al. 2019). For instance, the PhenoAge and GrimAge clocks were trained based on clinical and physiological phenotypes to capture aging‐related physiological deterioration and were associated with disease and mortality risk (Levine et al. 2018; Lu et al. 2019). Their principal component (PC)‐based versions, PCPhenoAge and PCGrimAge, were subsequently introduced to reduce technical noise from DNA methylation microarrays, demonstrating improved reliability in longitudinal settings (Higgins‐Chen et al. 2022). Another clock, DunedinPACE, was developed to measure dynamic aging rates by modeling changing rates of 19 biomarkers over two decades (Belsky et al. 2022).

A central debate about epigenetic clocks concerns the causal interpretation: whether epigenetic clocks should be viewed as causal biomarkers of biological aging, or merely as statistical proxies for time and accumulated physiological burden (Ferrucci et al. 2025; Ikram 2024). Several randomized controlled trials (RCTs) testing anti‐aging properties of lifestyle or pharmacological interventions have used epigenetic clocks as primary outcomes under the assumption that they are causal aging biomarkers (Waziry et al. 2023; Belsky et al. 2017; Yaskolka Meir et al. 2021; Chen et al. 2018). If this assumption holds, then interventions that alter the trajectory of epigenetic aging should also manifest in downstream physiological outcomes, such as measures of cardiometabolic health (e.g., glycemic traits, lipid profiles), given their associations with epigenetic clocks (Ammous et al. 2021; Miao et al. 2024; Lo and Lin 2022; Lin et al. 2024; Joyce et al. 2021). However, the extent to which the gerotherapeutic effects could be translated into prevention of physiological dysfunction remains an open question. Alternatively, if epigenetic clocks merely correlate with physiological measures, investigating how their associations with biomarkers shift in response to gerotherapeutic interventions may clarify the ultimate role of epigenetic clocks in geroscience.

Another important question is whether epigenetic clocks are generalizable across populations. The PhenoAge, GrimAge, and DunedinPACE were originally developed and validated in cohorts of the general population (Levine et al. 2018; Lu et al. 2019; Belsky et al. 2022), yet few studies have evaluated their utility in individuals with substantially different metabolic profiles, such as adults with obesity, a population with a high burden of cardiometabolic disease (Valenzuela et al. 2023). Understanding these relationships informs their broader clinical and research applications.

Using data from the Macronutrients and Heart Disease Risk (MACRO) trial, a 12‐month dietary weight‐loss intervention among adults with obesity (Bazzano et al. 2014), we assessed and compared the associations of three epigenetic clocks (PCPhenoAge acceleration, PCGrimAge acceleration, and DunedinPACE) with cardiometabolic biomarkers at pre‐intervention baseline and post‐intervention follow‐up. Leveraging the longitudinal design, we conducted both change‐to‐change analyses and mediation analyses to assess the potential causal relationships between these epigenetic aging measures and cardiometabolic biomarkers. Our findings may offer new insights into the biological interpretation and translational relevance of epigenetic clocks.

## 2 Methods

### 2.1 Study Population

The MACRO trial (clinicaltrial.gov identifier: NCT00609271) was a 12‐month, parallel‐arm randomized trial conducted from 2008 to 2011 at Tulane University Health Sciences Center, New Orleans, Louisiana. The trial's primary aim was to evaluate the effects of a low‐carbohydrate diet on body weight and cardiovascular risk factors compared to a low‐fat diet. After finishing the 12‐month follow‐up, the low‐carbohydrate diet led to greater weight loss and cardiovascular risk factor reduction than the low‐fat diet (Bazzano et al. 2014). Participants aged 22–75 years with a body mass index (BMI) of 30–45 kg/m^2^ were eligible for inclusion. Exclusion criteria included pregnancy, type 2 diabetes, cardiovascular disease (CVD), or chronic renal disease. A total of 148 participants were randomized to either a low‐carbohydrate (< 40 g/day of digestible carbohydrates, *N* = 75) or a low‐fat diet (< 30% energy from total fat, < 7% from saturated fat, *N* = 73), while neither diet included a specific calorie or energy goal. The original report of the trial reported high adherence based on 24‐h recalls and urinary ketone testing (Bazzano et al. 2014). Detailed information about the study design and dietary intervention has been described elsewhere and in the Supplemental Methods (Bazzano et al. 2014). All procedures were approved by the Institutional Review Board of Tulane University Health Sciences Center, and all participants provided written informed consent.

**FIGURE 1:**
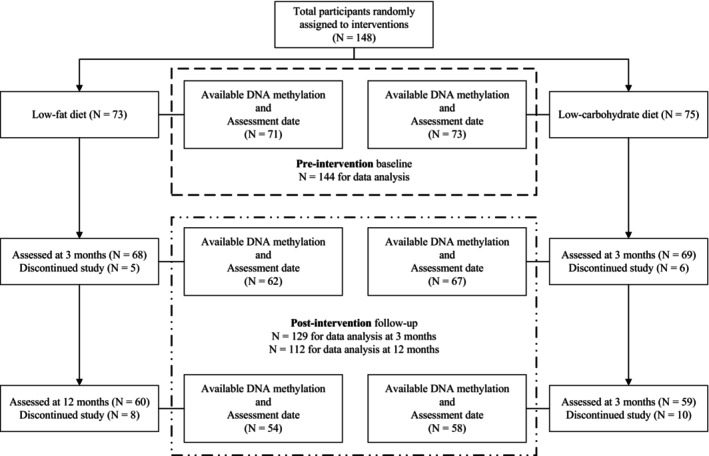
The study flowchart.

The study flow is presented in Figure 1. Of the 148 participants who were randomly assigned to either the low‐fat or low‐carbohydrate diet group, 144 had available DNA methylation data and assessment dates at screening visit and were included in the pre‐intervention cross‐sectional analysis. During post‐intervention periods, 129 participants (62 in low‐fat diet, and 67 in low‐carbohydrate diet) and 112 participants (54 in low‐fat diet, and 58 in low‐carbohydrate diet) were included for longitudinal analysis at 3 and 12 months, respectively. Reasons for attrition included discontinuation and lack of available biospecimens.

### 2.2 DNA Methylation and Epigenetic Clocks

DNA methylation profiling was conducted on 392 samples from 148 unique participants, with samples collected at baseline, 3 months, and 12 months. An additional five quality control samples provided by GenomeScan (GenomeScan B.v., Leiden, The Netherlands) were included, resulting in a total of 397 samples. To minimize technical batch effects and avoid systematic confounding by timepoint or intervention group, all samples were randomized prior to array processing. Each sample was randomly assigned a GenomeScan ID from 1 to 397. Samples were then allocated to five 96‐well plates based on this randomized order: Plate 1 included IDs 1–96, Plate 2 included 97–192, Plate 3 included 193–288, Plate 4 included 289–384, and Plate 5 included 385–397. In each plate, a variable position was reserved for a control sample.

Genomic DNA was bisulfite‐converted using the EZ DNA Methylation Kit (Zymo Research) and used for microarray‐based DNA methylation analysis, performed at GenomeScan and AIT (Austrian Institute of Technology GmbH, Vienna, Austria) on the Human Methylation850k BeadChip (Illumina Inc., San Diego, CA, USA). This array interrogates over 850,000 CpG sites representing about 99% of the RefSeq genes. The bisulfite‐converted DNA was processed and hybridized to the Human Methylation850 BeadChip (Illumina, inc.), according to the manufacturer's instructions. The BeadChip images were scanned on the iScan system, and the data quality was assessed using the R script MethylAid (van Iterson et al. 2014) using default analysis settings. All samples showed a detected CpG above 95%, > 807,500 detected CpG. Quality control and normalization were performed by GenomeScan. DNA methylation normalization on dye‐bias, background noise, and batch effects was performed using the preprocessFunnorm function from the *minfi* R package (Aryee et al. 2014). Immune cell composition (CD8^+^ T‐cells, CD4^+^ T‐cells, natural killer cells, B‐cells, monocytes and neutrophils) was estimated using the Houseman approach adapted to EPIC arrays using the *FlowSorted.Blood.EPIC* R package (Salas et al. 2018; Houseman et al. 2012). Beta values were then normalized for immune cell type composition using the *sva* R package (Leek et al. 2012), following the method described by Keller et al. (2020). The final beta values of DNA methylation ranged from 0–1 representing the percentage of methylated counts of total counts.

Three epigenetic clocks were calculated using the R package *dnaMethyAge* (Wang et al. 2024), including PCPhenoAge, PCGrimAge, and DunedinPACE. (Levine et al. 2018; Lu et al. 2019; Higgins‐Chen et al. 2022; Belsky et al. 2022) Epigenetic age acceleration was calculated as residuals from regressing PC clocks on chronological age, follow‐up time, and the interaction terms using linear mixed‐effect models. An epigenetic age acceleration value > 0 represented an accelerated aging process compared to chronological age. DunedinPACE, a direct measure of the aging rate, was also assessed, where values > 1 indicated accelerated aging.

### 2.3 Assessment of Covariates

The covariates included chronological age, sex, race/ethnicity, education levels, BMI, smoking status, alcohol use status, physical activity, total energy intake, and immune cell composition. The chronological age was calculated as the interval between the date of birth and the date of each visit. Sex was classified as men or women. Race/ethnicity was collected at baseline as Whites, Blacks, Asian, Hispanic, or Other. Highest education levels were recorded, including grade 11 or less, high school diploma, some college, degree from 2‐year college, degree from 4‐year college, some graduate school, and graduate degree. We further classified education levels into three categories: low (high school diploma or lower), medium (college graduation or lower), and high (graduate school or higher). Body weight and height were measured to the nearest 0.1 kg and 0.1 cm, using a single calibrated scale (Detecto, model 6855) and a wall‐mounted stadiometer at each visit. BMI was calculated as weight in kilograms divided by height in meters squared (kg/m^2^). Lifestyle factors (smoking, alcohol use, and physical activity) were collected at each visit. Participants were classified as ever smokers if they ever had > 100 cigarettes during their lifetime, and never smokers if not. Alcohol use information in the past 3 months at each visit was collected, and participants reported that alcohol use was classified as current alcohol users. Physical activity was calculated as the sum of hours of moderate to vigorous activities per week (walking, sports, dance, and conditioning) multiplied by each activity's individual metabolic equivalent value. Two 24‐h dietary recalls were obtained at each visit by a trained and certified staff member to reflect food composition, one on a weekday and another on a weekend. Total energy intake was then calculated based on food composition at each visit. We handled missing covariates with the missing indicators for categorical variables and mean imputation for continuous variables at each visit.

### 2.4 Assessment of Cardiometabolic Biomarkers

The cardiometabolic biomarkers included glycemic traits (glucose, insulin, homeostatic model assessment for insulin resistance [HOMA‐IR], triglyceride glucose [TyG] index), lipids (total cholesterol, high‐density lipoprotein cholesterol [HDL‐C], low‐density lipoprotein cholesterol [LDL‐C], triglycerides), C‐reactive protein (CRP), and hormones (adiponectin, ghrelin, peptide YY). Blood samples were collected after 12‐h fasting at baseline, 3‐month, and 12‐month follow‐up. Plasma glucose was measured using the Hitachi 902 Chemistry Analyzer and kits and reagents supplied by Hitachi‐Boehringer Mannheim. Plasma immunoreactive insulin was measured by radioimmunoassay kits (Linco Research, St. Charles, MO). HOMA‐IR, a measure of insulin resistance, was calculated as HOMA‐IR = (fasting insulin, uIU/mL) × (fasting glucose, mg/dL)/405 (Matthews et al. 1985). Another indicator of insulin resistance, TyG index, was estimated as TyG = ln[(fasting triglycerides, mg/dL) × (fasting glucose, mg/dL)/2] (Simental‐Mendía et al. 2008). Serum total cholesterol, HDL‐C, and triglyceride were measured according to enzymatic procedures (Hitachi 902 Chemistry Analyzer). LDL‐C was calculated using the Friedewald formula (Friedewald et al. 1972). Plasma CRP was measured by a high‐sensitivity latex‐enhanced immunonephelometric assay (Roche Diagnostics, Indianapolis, IN). Plasma adiponectin was measured by enzyme‐linked immunosorbent assay from R & D Systems, Minneapolis, MN. Appetite‐related hormones, peptide YY and ghrelin, were measured using radioimmunoassay methods (Millipore, Billerica, MA, and Linco Research, St. Charles, MO).

### 2.5 Statistical Analysis

We first summarized the characteristics of the study population at baseline (pre‐intervention screening), 3‐month, and 12‐month follow‐up visits. Dietary adherence was monitored throughout the study using multiple measures, including intake of total fat, saturated fat, and digestible carbohydrate intake (g/day), as well as the proportion of energy derived from fat, saturated fat, and carbohydrates. Cardiometabolic biomarkers were log‐transformed prior to downstream statistical analyses to improve normality.

We assessed the associations between epigenetic age acceleration and cardiometabolic biomarkers at pre‐intervention baseline and post‐intervention follow‐up, respectively. At baseline, we conducted cross‐sectional linear regression analyses, adjusting for baseline chronological age, sex, race (Whites, or non‐Whites), education levels (low, medium, or high), smoking status (ever, or never smokers), alcohol use (current, or non‐current users), physical activity, BMI, total energy intake, and immune cell composition (CD8^+^ T‐cells, CD4^+^ T‐cells, natural killer cells, B‐cells, monocytes and neutrophils). With longitudinal follow‐up data, we applied linear mixed‐effects models with a random intercept for each participant, adjusting for time, intervention group, and the same covariates to maintain consistency and comparability. Time‐varying covariates were updated at each visit. To investigate the dynamic coupling of epigenetic age acceleration and cardiometabolic biomarkers, we further modeled changes in epigenetic age acceleration (from baseline to 3‐ or 12‐month follow‐up) as independent variables and corresponding changes in cardiometabolic biomarkers as dependent variables, adjusting for baseline covariates and intervention group.

To evaluate the potential causal relationship between epigenetic age acceleration and cardiometabolic biomarkers, we performed mediation analysis to test whether weight loss (the primary goal of the MACRO trial)‐induced physiological improvements in cardiometabolic biomarkers could be attributed to changes in epigenetic aging, using the R package *mediation*. Among participants with both baseline and follow‐up data (12 months), change in BMI was modeled as the exposure, change in epigenetic age acceleration as the mediator, and change in each cardiometabolic biomarker as the outcome. We followed a two‐step method: first, we fitted the mediator model between the mediator and the exposure adjusting for baseline BMI, epigenetic age acceleration, and covariates; second, we fitted the outcome model between each outcome and the exposure further adjusting for the mediator and baseline levels of the outcome. A quasi‐Bayesian approximation was used to compute the 95% confidence intervals of the proportions of mediations with 1000 Monte Carlo draws.

We have conducted several sensitivity analyses. First, we tested interactions between epigenetic age acceleration and time to assess whether associations with cardiometabolic biomarkers changed over the course of the intervention. Second, we conducted stratified analyses by dietary groups (low‐carbohydrate vs. low‐fat) to explore potential differences in associations. Third, we visualized the trajectories of epigenetic age acceleration over time by dietary intervention group.

All statistical analyses were done with SAS version 9.4 (SAS Institute Inc. Cary, NC, USA) and R version 4.2.2. Model equations for each analysis were provided in the Supplemental Methods. Final effect estimates were back‐transformed from the log scale to represent percent changes with per‐unit difference in epigenetic age acceleration. To account for multiple comparisons, we considered a false discovery rate (FDR) correction (Glickman et al. 2014; Benjamini and Hochberg 1995), and a corrected FDR < 0.05 was considered statistically significant.

## 3 Results

### 3.1 Participant Characteristics

The characteristics of participants are shown in Table 1. At the pre‐intervention baseline visit, 50.7% were assigned to the low‐carbohydrate diet intervention, 88.9% were women, and the mean (SD) chronological age was 46.86 (10.25) years. The mean (SD) BMI was 35.44 (4.15) kg/m^2^, and the average total caloric intake exceeded 2000 kcal per day. As for lifestyle factors, 25.7% of participants smoked, and 57.0% were current alcohol users; the mean physical activity level was 19.79 MET/week. A total of 129 and 112 participants underwent the 3‐ and 12‐month follow‐up visits, respectively. Participants had lower energy intake and BMI after launching the dietary interventions, which was consistent with the goal of the trial. Participants reported healthier lifestyles at the 3‐month visit (e.g., non‐current alcohol use and more physical activity), which returned to near‐baseline levels by 12 months. The immune cell composition was relatively stable, with a slightly increasing trend for CD8^+^ T‐cells and a decreasing trend for B‐cells and monocytes over the study period.

**TABLE 1:** The characteristics of MACRO Trial participants by follow‐up time.

	Pre‐intervention baseline (*N* = 144)	Post‐intervention follow‐up	
		Three months (*N* = 129)	Twelve months (*N* = 112)
Dietary intervention, *n* (%)			
Low‐fat diets	71 (49.3%)	62 (48.1%)	54 (48.2%)
Low‐carbohydrate diets	73 (50.7%)	67 (51.9%)	58 (51.8%)
Chronological age, years	46.86 (10.25)	47.32 (10.01)	48.63 (9.74)
Women, *n* (%)	128 (88.9%)	114 (88.4%)	97 (86.6%)
Whites, *n* (%)	64 (44.4%)	57 (44.2%)	48 (42.9%)
Education, *n* (%)			
Low	7 (4.9%)	6 (4.7%)	5 (4.5%)
Medium	95 (66.0%)	86 (66.7%)	73 (65.2%)
High	42 (29.2%)	37 (28.7%)	34 (30.4%)
Ever smoking, *n* (%)	37 (25.7%)	33 (25.6%)	26 (23.4%)
Current alcohol use, *n* (%)	77 (57.0%)	50 (40.7%)	55 (52.4%)
Body mass index, kg/m^2^	35.44 (4.15)	34.07 (4.41)	34.19 (4.60)
Total energy intake, kcal	2012.64 (704.82)	1345.83 (440.94)	1488.97 (541.90)
Mean physical activity level, MET/week	19.79 (31.89)	25.86 (55.53)	16.41 (28.89)
Cardiometabolic biomarkers			
Fasting glucose, mg/dL	93.90 (10.06)	92.42 (9.65)	93.34 (11.48)
Fasting insulin, μIU/mL	14.96 (8.65)	12.42 (9.70)	12.07 (9.17)
HOMA‐IR	3.53 (2.23)	2.90 (2.37)	2.92 (3.03)
TyG index	8.48 (0.53)	8.39 (0.49)	8.33 (0.50)
Total cholesterol, mg/dL	201.63 (41.75)	198.22 (36.39)	201.32 (36.36)
Triglycerides, mg/dL	117.64 (68.44)	107.30 (61.09)	99.48 (53.74)
LDL cholesterol, mg/dL	122.81 (36.71)	122.24 (32.39)	120.57 (32.06)
HDL cholesterol, mg/dL	55.27 (13.22)	54.54 (13.64)	60.88 (16.86)
C‐reactive protein, mg/L	7950.61 (4997.62)	9102.76 (5284.10)	9893.87 (5663.78)
Adiponectin, μg/mL	4.81 (4.65)	5.10 (5.55)	5.00 (5.55)
Total ghrelin, pg/mL	825.09 (281.29)	822.01 (283.31)	784.26 (272.91)
Total peptide YY, pg/mL	114.90 (28.96)	108.61 (32.52)	74.99 (26.63)
Immune cell composition, %			
CD8^+^ T‐cells	12.46 (4.77)	12.56 (5.14)	12.62 (5.16)
CD4^+^ T‐cells	22.45 (6.01)	23.54 (6.72)	23.21 (6.73)
Natural killer cells	0.05 (0.02)	0.05 (0.02)	0.05 (0.02)
B‐cells	7.99 (3.07)	7.67 (2.93)	7.51 (2.86)
Monocytes	9.95 (3.18)	9.89 (2.99)	9.64 (2.77)
Neutrophils	43.95 (10.64)	42.92 (10.43)	43.20 (11.47)
Epigenetic clocks			
PCPhenoAge, years	46.11 (8.64)	46.30 (8.42)	47.26 (7.65)
PCGrimAge, years	57.89 (7.97)	58.24 (7.84)	59.28 (7.51)
DunedinPACE	1.00 (0.10)	1.00 (0.11)	0.99 (0.10)

*Note:* Values are means (SD) for continuous variables; n (percentage) for categorical variables.

Abbreviations: HDL, high‐density lipoprotein; HOMA‐IR, homeostatic model assessment for insulin resistance; LDL, low‐density lipoprotein.

As shown in Supplemental Table 1, participants in the low‐fat diet group substantially reduced their intake of total and saturated fat by 3 months, with corresponding reductions in the proportion of energy from fat (27.35%) and saturated fat (7.98%), close to the intervention goal. Conversely, participants in the low‐carbohydrate diet group exhibited a marked decrease in digestible carbohydrate intake, with mean intake reduced from 223.12 (89.31) g at baseline to 82.52.70 (43.31) g at 3 months, although they did not reach the original goal of < 40 g/day. These patterns indicate that both groups adhered to their assigned macronutrient goals.

The correlation coefficients were ≥ 0.78 between PCPhenoAge and chronological age, and ≥ 0.96 between PCGrimAge and chronological age at both pre‐ and post‐intervention visits (Supplemental Table 2). Three measures of epigenetic age acceleration had poor correlations with chronological age across all visits.

### 3.2 Associations Between Epigenetic Age Acceleration and Cardiometabolic Biomarkers

**FIGURE 2:**
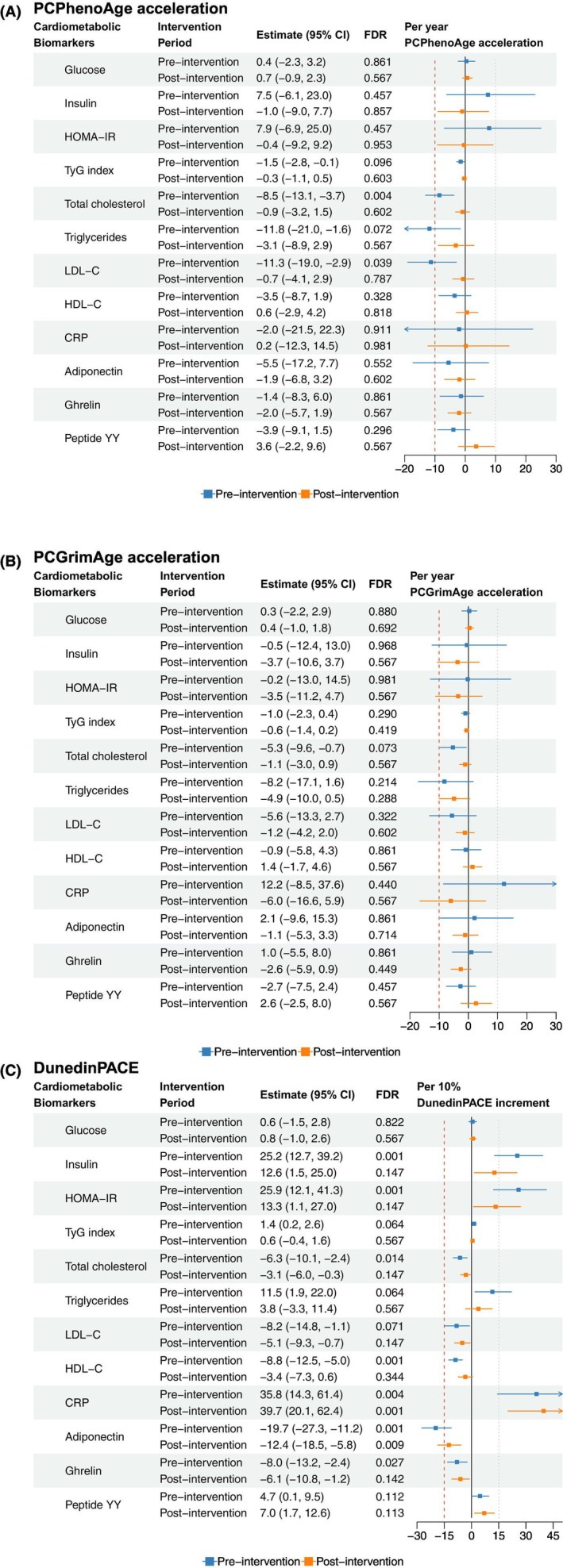
The associations between epigenetic age acceleration metrics and cardiometabolic biomarkers at pre‐ and post‐intervention periods. (A) PCPhenoAge acceleration; (B) PCGrimAge acceleration; (C) DunedinPACE. CRP, C‐reactive protein; HDL‐C, high‐density lipoprotein cholesterol; HOMA‐IR, homeostatic model assessment for insulin resistance; LDL‐C, low‐density lipoprotein cholesterol. Pre‐intervention linear models were adjusted for baseline levels of chronological age, sex, race, education, smoking, alcohol use, body mass index, total energy intake, physical activity, and immune cell composition. Post‐intervention linear mixed‐effects models were adjusted for chronological age, sex, race, education, smoking, alcohol use, concurrent body mass index, total energy intake, physical activity, immune cell composition, time, and intervention groups.

Figure 2 presents the association between epigenetic age acceleration and cardiometabolic biomarkers. At baseline, each one‐year increment of PCPhenoAge acceleration was significantly associated with lower total cholesterol (−8.5%; 95% confidence interval [CI]: −13.1% to −3.7%) and LDL‐C (−11.3%; 95% CI: −19.0% to −2.9%), both FDR < 0.05. Higher DunedinPACE (per 10% increase) was associated with higher levels of insulin (25.2%; 95% CI: 12.7%–39.2%), HOMA‐IR (25.9%; 95% CI: 12.1%–41.3%), and CRP (35.8%; 95% CI: 14.3%–61.4%), as well as lower levels of total cholesterol (−6.3%; 95% CI: −10.1% to −2.4%), HDL‐C (−8.8%; 95% CI: −12.5% to −5.0%), adiponectin (−19.7%; 95% CI: −27.3% to −11.2%), and ghrelin (−8.0%; 95% CI: −13.2% to −2.4%); all associations were statistically significant (FDR < 0.05). In response to dietary interventions, only the associations between DunedinPACE and CRP and adiponectin remained statistically significant (FDR < 0.05). Specifically, a 10% higher DunedinPACE was associated with a 39.7% higher CRP (95% CI: 20.1%–62.4%) and a 12.4% lower adiponectin (95% CI: −18.5% to −5.5%). These associations were consistent across both the 3‐ and 12‐month follow‐up visits, with no significant interactions by time (FDR for interaction > 0.05; Table S3) or by dietary intervention group (FDR for interaction > 0.05; Table S4). When modeling the dynamic changes in epigenetic age acceleration and corresponding changes in cardiometabolic biomarkers over time, no statistically significant associations were observed (FDR > 0.05; Table 2).

**TABLE 2:** The associations between changes of epigenetic age acceleration and changes of cardiometabolic biomarkers.

Epigenetic age acceleration metrics	Cardiometabolic biomarkers	Three months^a^		Twelve months^a^	
		Estimates (95% CI)	FDR	Estimates (95% CI)	FDR
PCPhenoAge acceleration	Glucose	−0.4 (−1.9 to 1.2)	0.903	1.1 (−0.9 to 3.1)	0.954
	Insulin	−1.7 (−9.1 to 6.3)	0.903	3.7 (−4.9 to 13.0)	0.954
	HOMA‐IR	−2.2 (−10.4 to 6.8)	0.903	4.8 (−5.2 to 15.8)	0.954
	TyG index	−0.6 (−1.2–0)	0.390	−0.4 (−1.2 to 0.4)	0.954
	Total cholesterol	−2.2 (−4.1 to −0.2)	0.390	−0.6 (−2.3 to 1.2)	0.981
	Triglycerides	−4.1 (−9.1 to 1.1)	0.390	−4.3 (−9.2 to 0.9)	0.954
	LDL‐C	−3.8 (−7.2 to −0.4)	0.390	−1.2 (−5.0 to 2.8)	0.981
	HDL‐C	0.1 (−2.8 to 3.1)	0.972	1.2 (−1.7 to 4.2)	0.954
	CRP	1.8 (−7.0 to 11.4)	0.903	3.9 (−6.9 to 15.9)	0.981
	Adiponectin	0.3 (−2.6 to 3.3)	0.968	0.6 (−3.5 to 4.8)	0.981
	Ghrelin	−1.0 (−4.4 to 2.6)	0.903	−0.1 (−3.4 to 3.3)	0.981
	Peptide YY	−2.8 (−6.7 to 1.3)	0.506	0.5 (−3.4 to 4.5)	0.981
PCGrimAge acceleration	Glucose	0.3 (−1.1 to 1.7)	0.903	0 (−1.9 to 2.0)	0.981
	Insulin	−0.4 (−7.2 to 6.9)	0.972	1.1 (−7.2 to 10.2)	0.981
	HOMA‐IR	−0.2 (−7.7 to 7.9)	0.972	1.1 (−8.3 to 11.5)	0.981
	TyG index	−0.5 (−1.1 to 0.1)	0.390	−0.5 (−1.1 to 0.1)	0.954
	Total cholesterol	−2.1 (−3.8 to −0.3)	0.390	−1.9 (−3.6 to −0.1)	0.954
	Triglycerides	−4.6 (−9.0 to 0)	0.390	−4.4 (−9.3 to 0.8)	0.954
	LDL‐C	−2.2 (−5.4 to 1.1)	0.506	−2.3 (−6.0 to 1.6)	0.954
	HDL‐C	−0.5 (−3.0 to 2.1)	0.903	1.8 (−1.1 to 4.9)	0.954
	CRP	0.9 (−6.7 to 9.1)	0.955	−1.1 (−11.4 to 10.4)	0.981
	Adiponectin	−0.6 (−3.3 to 2.2)	0.903	0.2 (−3.8 to 4.4)	0.981
	Ghrelin	−1.8 (−4.8–1.3)	0.584	0.4 (−2.9 to 3.8)	0.981
	Peptide YY	−2.4 (−5.8 to 1.1)	0.506	−0.4 (−4.4 to 3.8)	0.981
DunedinPACE^b^	Glucose	0.1 (−3.6 to 3.9)	0.972	0.1 (−4.1 to 4.5)	0.981
	Insulin	−9.5 (−24.3 to 8.2)	0.584	3.7 (−14.8 to 26.1)	0.981
	HOMA‐IR	−9.5 (−25.9 to 10.5)	0.625	3.8 (−17.0 to 29.8)	0.981
	TyG index	−1.3 (−2.8 to 0.3)	0.390	−0.8 (−2.3 to 0.8)	0.954
	Total cholesterol	−3.5 (−7.8 to 0.9)	0.390	−1.7 (−5.7 to 2.4)	0.954
	Triglycerides	−9.8 (−20.0 to 1.7)	0.390	−6.4 (−16.9 to 5.5)	0.954
	LDL‐C	−2.6 (−10.4 to 6.0)	0.903	−1.7 (−10.2 to 7.6)	0.981
	HDL‐C	2.8 (−3.6 to 9.7)	0.728	5.1 (−1.7 to 12.4)	0.954
	CRP	18.6 (−2.7 to 44.6)	0.390	11.3 (−13.1 to 42.5)	0.954
	Adiponectin	−0.9 (−7.5 to 6.1)	0.955	−0.7 (−9.4 to 8.9)	0.981
	Ghrelin	4.4 (−3.7 to 13.1)	0.597	1.7 (−5.4 to 9.4)	0.981
	Peptide YY	−5.2 (−13.5 to 4.0)	0.584	−1.7 (−10.0 to 7.4)	0.981

Abbreviations: CRP, C‐reactive protein; FDR, false discovery rate; HDL‐C, high‐density lipoprotein cholesterol; HOMA‐IR, homeostatic model assessment for insulin resistance; LDL‐C, low‐density lipoprotein cholesterol.

^a^ Linear mixed‐effects models adjusted for baseline levels of chronological age, sex, race, education, smoking, alcohol use, body mass index, total energy intake, physical activity, immune cell composition, and intervention groups.

^b^ Per 0.1 unit increment.

### 3.3 The Mediation Role of Epigenetic Age Acceleration in Weight Loss‐Induced Changes in Measure of Cardiometabolic Health

Trajectories of epigenetic age acceleration over the study period are presented in Supplemental Figure 1. Between baseline and the 3‐month visit, little to no change was observed in any of the epigenetic age acceleration metrics. By the 12‐month visit, participants in the low‐carbohydrate diet group exhibited higher PCPhenoAge and PCGrimAge acceleration compared to those in the low‐fat diet group. In contrast, DunedinPACE slightly declined from the baseline in both dietary groups. Based on these trends, we focused our mediation analyses on the 12‐month changes in epigenetic age acceleration.

During the 12‐month follow‐up period, dietary intervention‐induced weight loss was significantly associated with concurrent changes in several cardiometabolic biomarkers (Table 3). Changes in BMI were positively correlated with changes in glucose, insulin, HOMA‐IR, TyG index, triglycerides, and CRP, and negatively associated with changes in adiponectin and ghrelin. However, mediation analyses revealed that none of these associations were significantly mediated by concurrent changes in any epigenetic age acceleration measure. Specifically, all indirect effects and proportions mediated were non‐significant (FDR > 0.05), suggesting that the observed associations were not driven through changes in epigenetic aging.

**TABLE 3:** Mediation analysis for 12‐month changes of BMI, epigenetic age acceleration, and cardiometabolic biomarkers.

Mediators	Outcomes	Total effect		Direct effect		Indirect effect		Proportional mediated, %	
		Estimates (95% CI)	FDR	Estimates (95% CI)	FDR	Estimates (95% CI)	FDR	Estimates (95% CI)	FDR
PCPhenoAge acceleration	Glucose	**0.014** (**0.004–0.024**)	**0.004**	**0.014** (**0.004** to **0.024**)	**0.004**	0 (−0.001 to 0.002)	0.97	0.5 (−14.4 to 15)	0.99
	Insulin	**0.121** (**0.077–0.164**)	**< 0.001**	**0.12** (**0.077** to **0.164**)	**< 0.001**	0.001 (−0.005 to 0.007)	0.97	0.1 (−4.8 to 6.2)	0.99
	HOMA‐IR	**0.135** (**0.089–0.183**)	**< 0.001**	**0.135** (**0.087** to **0.183**)	**< 0.001**	0.001 (−0.006 to 0.009)	0.97	0.3 (−5.4 to 6.7)	0.99
	TyG index	**0.008** (**0.004–0.011**)	**< 0.001**	**0.008** (**0.004** to **0.011**)	**< 0.001**	0 (0 to 0)	0.98	0 (−7 to 5.6)	0.99
	Total cholesterol	0.003 (−0.006 to 0.012)	0.661	0.003 (−0.006 to 0.012)	0.659	0 (−0.003 to 0.002)	0.97	−0.5 (−212.2 to 184.6)	0.99
	Triglycerides	**0.049** (**0.02–0.079**)	**0.007**	**0.049** (**0.02–0.079**)	**0.007**	0 (−0.004 to 0.003)	0.97	0 (−9.8 to 7)	0.99
	LDL‐C	0.002 (−0.015 to 0.019)	0.926	0.002 (−0.014 to 0.019)	0.907	−0.001 (−0.005 to 0.003)	0.97	2.8 (−291.4 to 266.5)	0.99
	HDL‐C	−0.006 (−0.025 to 0.012)	0.661	−0.007 (−0.024 to 0.012)	0.659	0 (−0.003 to 0.004)	0.97	−0.1 (−127.6 to 139.3)	0.99
	CRP	**0.105** (**0.042–0.166**)	**< 0.001**	**0.104** (**0.042–0.165**)	**< 0.001**	0.001 (−0.008 to 0.013)	0.97	0.3 (−9.6 to 13.4)	0.99
	Adiponectin	**−0.062** (**−0.082** to **‐0.042**)	**< 0.001**	**−0.062** (**−0.082** to **−0.042**)	**< 0.001**	0 (−0.003 to 0.004)	0.97	−0.2 (−7.3 to 4.1)	0.99
	Ghrelin	**−0.027** (**−0.046** to **−0.008**)	**0.009**	**−0.027** (**−0.045** to **−0.008**)	**0.009**	0 (−0.003 to 0.003)	0.97	0.2 (−13 to 13.1)	0.99
	Peptide YY	0.002 (−0.021 to 0.026)	0.926	0.002 (−0.022–0.025)	0.944	0.001 (−0.004 to 0.006)	0.97	2 (−186.9 to 192.5)	0.99
PCGrimAge acceleration	Glucose	**0.014** (**0.004–0.024**)	**0.004**	**0.014** (**0.004** to **0.024**)	**< 0.001**	0 (−0.002 to 0.001)	0.97	−0.2 (−17.8 to 11.6)	0.99
	Insulin	**0.122** (**0.076–0.166**)	**< 0.001**	**0.123** (**0.077** to **0.168**)	**< 0.001**	−0.001 (−0.01 to 0.006)	0.97	−0.6 (−10.1 to 4.5)	0.99
	HOMA‐IR	**0.136** (**0.085–0.184**)	**< 0.001**	**0.137** (**0.086** to **0.186**)	**< 0.001**	−0.001 (−0.01 to 0.006)	0.97	−0.4 (−8.7 to 3.9)	0.99
	TyG index	**0.008** (**0.004–0.011**)	**< 0.001**	**0.008** (**0.004** to **0.011**)	**< 0.001**	0 (0–0.001)	0.97	0.8 (−6 to 12.3)	0.99
	Total cholesterol	0.003 (−0.007 to 0.013)	0.698	0.001 (−0.008 to 0.01)	0.907	0.001 (−0.001 to 0.005)	0.97	19 (−319.5 to 411.9)	0.99
	Triglycerides	**0.049** (**0.021–0.078**)	**< 0.001**	**0.048** (**0.021** to **0.076**)	**< 0.001**	0.001 (−0.003 to 0.006)	0.97	0.7 (−8.5 to 14.5)	0.99
	LDL‐C	0.001 (−0.017–0.019)	0.926	−0.001 (−0.019 to 0.017)	0.944	0.002 (−0.002 to 0.008)	0.97	7.2 (−482.6–495.8)	0.99
	HDL‐C	−0.007 (−0.024 to 0.01)	0.661	−0.006 (−0.024 to 0.011)	0.685	−0.001 (−0.004 to 0.002)	0.97	1.7 (−122 to 145.8)	0.99
	CRP	**0.104** (**0.039–0.167**)	**< 0.001**	**0.106** (**0.041** to **0.169**)	**< 0.001**	−0.002 (−0.014 to 0.007)	0.97	−1 (−18.7 to 7.9)	0.99
	Adiponectin	**−0.062** (**−0.08** to **−0.042**)	**< 0.001**	**−0.062** (**−0.082** to **‐0.042**)	**< 0.001**	0.001 (−0.002 to 0.005)	0.97	−0.5 (−7.7 to 3.4)	0.99
	Ghrelin	**−0.028** (**−0.046** to **‐0.01**)	**0.007**	**−0.029** (**−0.047** to **‐0.011**)	**0.007**	0.001 (−0.001 to 0.006)	0.97	−3.4 (−31.3 to 5.7)	0.99
	Peptide YY	0.001 (−0.023 to 0.025)	0.926	0.003 (−0.021 to 0.026)	0.907	−0.001 (−0.007 to 0.002)	0.97	0.2 (−272.7 to 201)	0.99
DunedinPACE	Glucose	**0.013** (**0.004–0.023**)	**0.009**	**0.013** (**0.004** to **0.022**)	**0.009**	0 (−0.002 to 0.001)	0.97	−0.6 (−19.5 to 8.7)	0.99
	Insulin	**0.118** (**0.073–0.163**)	**< 0.001**	**0.119** (**0.075** to **0.164**)	**< 0.001**	−0.001 (−0.009 to 0.006)	0.97	−0.5 (−9.1 to 4.3)	0.99
	HOMA‐IR	**0.132** (**0.081–0.181**)	**< 0.001**	**0.133** (**0.082** to **0.181**)	**< 0.001**	−0.001 (−0.01 to 0.005)	0.97	−0.4 (−8.7 to 3.8)	0.99
	TyG index	**0.008** (**0.004–0.011**)	**< 0.001**	**0.008** (**0.004** to **0.011**)	**< 0.001**	0 (0–0)	0.97	0.1 (−6.2 to 7.2)	0.99
	Total cholesterol	0.003 (−0.008 to 0.012)	0.698	0.003 (−0.008 to 0.012)	0.75	0 (−0.001 to 0.002)	0.97	1.3 (−85.4 to 114.5)	0.99
	Triglycerides	**0.05** (**0.021–0.08**)	**0.004**	**0.049** (**0.022** to **0.079**)	**0.004**	0 (−0.003 to 0.005)	0.97	0.3 (−9.6 to 10.1)	0.99
	LDL‐C	0.001 (−0.017 to 0.018)	0.994	0 (−0.018 to 0.017)	0.998	0 (−0.002 to 0.003)	0.97	0 (−151.9 to 139.3)	0.99
	HDL‐C	−0.007 (−0.025 to 0.011)	0.661	−0.006 (−0.024 to 0.012)	0.697	−0.001 (−0.006 to 0.002)	0.97	5.1 (−172.3 to 171.1)	0.99
	CRP	**0.105** (**0.042–0.168**)	**< 0.001**	**0.108** (**0.047** to **0.168**)	**< 0.001**	−0.002 (−0.018 to 0.009)	0.97	−1.1 (−23.1 to 9)	0.99
	Adiponectin	**−0.062** (**−0.082** to **‐0.042**)	**< 0.001**	**−0.063** (**−0.082** to **‐0.043**)	**< 0.001**	0 (−0.002 to 0.004)	0.97	−0.2 (−6.6 to 3.8)	0.99
	Ghrelin	**−0.029** (**−0.047** to **−0.012**)	**0.004**	**−0.029** (**−0.047** to **‐0.012**)	**0.004**	0 (−0.003 to 0.002)	0.97	0.2 (−8.5 to 10.8)	0.99
	Peptide YY	0.001 (−0.023 to 0.024)	0.926	0.001 (−0.023 to 0.024)	0.944	0 (−0.003 to 0.004)	0.97	0.1 (−126.2 to 167.2)	0.99

*Note:* The mediator models were between the mediator and the exposure adjusting for baseline levels of chronological age, sex, race, education, smoking, alcohol use, body mass index, total energy intake, physical activity, immune cell composition, intervention groups, and baseline epigenetic age acceleration. The outcome models between each outcome and the exposure further adjusting for the mediator and baseline levels of the outcome.

Abbreviations: CRP, C‐reactive protein; FDR, false discovery rate; HDL‐C, high‐density lipoprotein cholesterol; HOMA‐IR, homeostatic model assessment for insulin resistance; LDL‐C, low‐density lipoprotein cholesterol.

## 4 Discussion

In this randomized trial of dietary interventions among adults with obesity, we investigated how epigenetic aging relates to a broad spectrum of cardiometabolic biomarkers before and after weight‐loss interventions. At baseline, DunedinPACE was significantly associated with multiple markers of metabolic and inflammatory health, including higher insulin, HOMA‐IR, and CRP, and lower HDL‐C, adiponectin, and ghrelin. In contrast, PCPhenoAge acceleration showed only modest associations with cholesterol measures, and PCGrimAge acceleration was not significantly related to any biomarker. Following the dietary interventions, most of the baseline associations of DunedinPACE were attenuated, with only CRP and adiponectin remaining significant, suggesting that weight loss might weaken the phenotypic expression of accelerated aging on cardiometabolic profiles. However, we did not observe significant associations between changes in epigenetic age acceleration and changes in cardiometabolic measures over time, nor evidence that epigenetic aging mediates weight loss‐induced changes in cardiometabolic biomarkers.

Our findings contribute to the central discussion in epigenetic aging research: whether accelerated epigenetic aging serves as a causal and modifiable pathway linking lifestyle factors and health outcomes or merely reflects underlying physiological differences. Recently, epigenetic clocks have been proposed as a target for longevity‐promoting intervention (Perri et al. 2024; Moqri et al. 2023), whereas evidence supporting its substantial improvements in health outcomes remains sparse, compared with other aging biomarkers that reflect directly physiological, functional, or cognitive domains (e.g., grip strength, blood pressure) (Perri et al. 2024). In the study, DunedinPACE demonstrated robust cross‐sectional associations with a range of cardiometabolic biomarkers, particularly those related to insulin resistance, inflammation, and adipose‐derived hormones. However, despite significant weight loss and corresponding improvements in cardiometabolic biomarkers over 12 months (Bazzano et al. 2014), these associations were substantially attenuated following dietary interventions. This pattern suggests a potential uncoupling between epigenetic aging and short‐term cardiometabolic changes, as demonstrated by non‐significant longitudinal associations between changes in epigenetic aging and changes in biomarkers. Moreover, mediation analyses did not support epigenetic age acceleration as a pathway through which weight loss led to cardiometabolic improvement. These findings suggest that while DunedinPACE is designed to reflect the pace of aging, it may not fully capture rapid physiological changes induced by lifestyle interventions over a 12‐month period. Instead, it may be more indicative of cumulative biological burden that changes gradually over time, and thus, may function more reliably as indicators of long‐term biological aging rather than as measures of short‐term health improvements. Therefore, the clinical use of these measures as surrogate endpoints for intervention efficacy should remain exploratory and be interpreted with more established physiological markers. Further research is needed to clarify the temporal dynamics and sensitivity of these clocks to interventions targeting healthy longevity.

Our study confirms previously observed associations between epigenetic clocks and cardiometabolic biomarkers and extends their utility to a high‐risk population with obesity. For example, we observed inverse associations between both PCPhenoAge and DunedinPACE with lipid profiles, consistent with findings from a nationally representative cohort of older U.S. adults (Lin et al. 2024). Moreover, we found that DunedinPACE may outperform other epigenetic aging measures in evaluating cardiometabolic health, aligning with findings from 1070 middle‐aged Chinese twins, where more temporal associations with glycemic traits were observed for DunedinPACE than for other epigenetic clocks (Miao et al. 2024). Our findings reinforce these observations, extending them to a broader range of cardiometabolic biomarkers, covering glycemic traits, lipid profiles, inflammation and hormones, and demonstrating relevance in adults with obesity. In contrast, we found fewer associations involving PCPhenoAge or PCGrimAge, a pattern consistent with the CALERIE trial, where caloric restriction slowed DunedinPACE but had limited effects on PCPhenoAge and PCGrimAge (Waziry et al. 2023). This discrepancy likely reflects differences in the biological processes each measure captures. While PCPhenoAge and PCGrimAge were trained to predict morbidity and mortality based on cross‐sectional clinical biomarker data (Levine et al. 2018; Lu et al. 2019), DunedinPACE was trained to capture the pace of biological deterioration over time (Belsky et al. 2022), potentially making it more responsive to various cardiometabolic biomarkers. It is noteworthy that the associations between DunedinPACE and CRP and adiponectin remained significant after interventions, suggesting its potential utility in capturing inflammation‐ and hormone‐related aspects of cardiometabolic health.

Although the primary results of the MACRO trial indicated that the low‐carbohydrate diet led to greater reductions in certain cardiovascular risk factors compared to the low‐fat diet (Bazzano et al. 2014), our findings suggest that these differences did not translate into divergent changes in epigenetic aging measures over the 12‐month intervention. The trajectories of epigenetic age acceleration support the observed uncoupling between epigenetic aging and short‐term changes in cardiometabolic biomarkers. Interestingly, we observed a slight increase in PCPhenoAge and PCGrimAge acceleration among participants in the low‐carbohydrate group relative to the low‐fat group. One possible explanation lies in the intervention target of the low‐carbohydrate arm (< 40 g/day digestible carbohydrate), as prior research has linked very low‐carbohydrate diets to increased mortality risk (Mazidi et al. 2019), despite their benefits for insulin sensitivity (Samaha et al. 2003), fat oxidation (Hyde et al. 2019), and appetite modification (Hu et al. 2016). In contrast, the patterns of DunedinPACE and epigenetic aging measures of the low‐fat diet group were comparable to results of the CALERIE trial where caloric restriction slowed DunedinPACE (Waziry et al. 2023). These observations may indicate that reduction in energy intake, rather than macronutrient composition, plays a more central role in modulating biological aging. It is also possible that macronutrient‐specific effects on cardiometabolic health occur through mechanisms not fully captured by epigenetic clocks, further highlighting their potential limitations in detecting short‐term physiological responses.

Our study has several strengths. First, by leveraging repeated measures of both epigenetic aging and a comprehensive set of cardiometabolic biomarkers within a randomized trial setting, we were able to evaluate both cross‐sectional and longitudinal associations, providing clues for causal relationships and a more dynamic view of how epigenetic clocks respond to lifestyle intervention. Second, our focus on obese adults extends the generalizability of epigenetic clocks beyond originally general cohorts. Third, we were able to directly compare multiple epigenetic clocks in capturing physiological aging processes, highlighting DunedinPACE as a potentially more responsive measure. However, several limitations should be acknowledged. The study population was relatively small, predominantly female, and free of chronic diseases, which may limit generalizability to broader populations with greater demographic and clinical diversity. In addition, the follow‐up duration of 12 months may have been insufficient to observe slower, cumulative changes in epigenetic clocks, particularly if these clocks reflect long‐term physiological burdens rather than short‐term responsiveness. Moreover, the trial did not include an untreated or ad libitum control group, which limits the ability to compare dietary interventions against usual dietary habits in the general population. Lastly, our study may not fully capture other dimensions of health (e.g., cognitive or physical function) that are relevant to aging and may be more tightly coupled with epigenetic aging trajectories. Future studies with longer follow‐up, more diverse populations, and integration of additional aging domains are needed to further clarify the role of epigenetic clocks in longevity‐promoting interventions.

In a 12‐month weight‐loss dietary intervention trial among adults with obesity, we found robust baseline associations between DunedinPACE and cardiometabolic biomarkers, particularly those related to insulin resistance, inflammation, and adipose‐derived hormones. However, these associations were attenuated following intervention, and we found no longitudinal evidence linking changes in epigenetic aging to changes in cardiometabolic biomarkers, nor support for a mediating role of epigenetic clocks. These findings raise caution in interpreting epigenetic clocks, particularly in the context of short‐term interventions, as causal mechanisms driving healthy aging. While epigenetic clocks like DunedinPACE may reflect accumulated physiological burden, their responsiveness to lifestyle changes may be more limited than previously assumed.

## Author Contributions

**Minghao Kou:** conceptualization, data curation, formal analysis, investigation, methodology, software, visualization, writing – original draft, writing – review and editing. **Xiang Li:** data curation, writing – review and editing. **Yoriko Heianza:** writing – review and editing. **Kirsten Dorans:** writing – review and editing. **Lydia Bazzano:** resources, writing – review and editing. **Lu Qi:** conceptualization, funding acquisition, project administration, resources, supervision, writing – review and editing.

## Ethics Statement

All procedures were approved by the Institutional Review Board of Tulane University Health Sciences Center and all participants provided written informed consent.

## Conflicts of Interest

The authors declare no conflicts of interest.

## Supporting information


**Data S1:** acel70224‐sup‐0001‐DataS1.docx.

## Data Availability

Data available on request due to privacy/ethical restrictions.

## References

[acel70224-bib-0001] Ammous, F. , W. Zhao , S. M. Ratliff , et al. 2021. “Epigenetic Age Acceleration Is Associated With Cardiometabolic Risk Factors and Clinical Cardiovascular Disease Risk Scores in African Americans.” Clinical Epigenetics 13, no. 1: 55. 10.1186/s13148-021-01035-3. 33726838 PMC7962278

[acel70224-bib-0002] Aryee, M. J. , A. E. Jaffe , H. Corrada‐Bravo , et al. 2014. “Minfi: A Flexible and Comprehensive Bioconductor Package for the Analysis of Infinium DNA Methylation Microarrays.” Bioinformatics 30, no. 10: 1363–1369. 10.1093/bioinformatics/btu049. 24478339 PMC4016708

[acel70224-bib-0003] Bazzano, L. A. , T. Hu , K. Reynolds , et al. 2014. “Effects of Low‐Carbohydrate and Low‐Fat Diets: A Randomized Trial.” Annals of Internal Medicine 161, no. 5: 309–318. 10.7326/m14-0180. 25178568 PMC4428290

[acel70224-bib-0004] Belsky, D. W. , A. Caspi , D. L. Corcoran , et al. 2022. “DunedinPACE, a DNA Methylation Biomarker of the Pace of Aging.” eLife 11: e73420. 10.7554/eLife.73420. 35029144 PMC8853656

[acel70224-bib-0005] Belsky, D. W. , K. M. Huffman , C. F. Pieper , I. Shalev , and W. E. Kraus . 2017. “Change in the Rate of Biological Aging in Response to Caloric Restriction: CALERIE Biobank Analysis.” Journals of Gerontology. Series A, Biological Sciences and Medical Sciences 73, no. 1: 4–10. 10.1093/gerona/glx096. 28531269 PMC5861848

[acel70224-bib-0006] Benjamini, Y. , and Y. Hochberg . 1995. “Controlling the False Discovery Rate: A Practical and Powerful Approach to Multiple Testing.” Journal of the Royal Statistical Society. Series B, Statistical Methodology 57, no. 1: 289–300.

[acel70224-bib-0007] Chen, L. , Y. Dong , J. Bhagatwala , A. Raed , Y. Huang , and H. Zhu . 2018. “Effects of Vitamin D3 Supplementation on Epigenetic Aging in Overweight and Obese African Americans With Suboptimal Vitamin D Status: A Randomized Clinical Trial.” Journals of Gerontology, Series A: Biological Sciences and Medical Sciences 74, no. 1: 91–98. 10.1093/gerona/gly223. PMC6612014 30256915

[acel70224-bib-0008] Elliott, M. L. , A. Caspi , R. M. Houts , et al. 2021. “Disparities in the Pace of Biological Aging Among Midlife Adults of the Same Chronological Age Have Implications for Future Frailty Risk and Policy.” Nature Aging 1, no. 3: 295–308. 10.1038/s43587-021-00044-4. 33796868 PMC8009092

[acel70224-bib-0009] Ferrucci, L. , N. Barzilai , D. W. Belsky , and V. N. Gladyshev . 2025. “How to Measure Biological Aging in Humans.” Nature Medicine 31, no. 4: 1057. 10.1038/s41591-025-03550-9. PMC12380361 40011691

[acel70224-bib-0010] Fransquet, P. D. , J. Wrigglesworth , R. L. Woods , M. E. Ernst , and J. Ryan . 2019. “The Epigenetic Clock as a Predictor of Disease and Mortality Risk: A Systematic Review and Meta‐Analysis.” Clinical Epigenetics 11, no. 1: 62. 10.1186/s13148-019-0656-7. 30975202 PMC6458841

[acel70224-bib-0011] Friedewald, W. T. , R. I. Levy , and D. S. Fredrickson . 1972. “Estimation of the Concentration of Low‐Density Lipoprotein Cholesterol in Plasma, Without Use of the Preparative Ultracentrifuge.” Clinical Chemistry 18, no. 6: 499–502. 4337382

[acel70224-bib-0012] Glickman, M. E. , S. R. Rao , and M. R. Schultz . 2014. “False Discovery Rate Control Is a Recommended Alternative to Bonferroni‐Type Adjustments in Health Studies.” Journal of Clinical Epidemiology 67, no. 8: 850–857. 10.1016/j.jclinepi.2014.03.012. 24831050

[acel70224-bib-0013] Higgins‐Chen, A. T. , K. L. Thrush , Y. Wang , et al. 2022. “A Computational Solution for Bolstering Reliability of Epigenetic Clocks: Implications for Clinical Trials and Longitudinal Tracking.” Nature Aging 2, no. 7: 644–661. 10.1038/s43587-022-00248-2. 36277076 PMC9586209

[acel70224-bib-0014] Horvath, S. , and K. Raj . 2018. “DNA Methylation‐Based Biomarkers and the Epigenetic Clock Theory of Ageing.” Nature Reviews. Genetics 19, no. 6: 371–384. 10.1038/s41576-018-0004-3. 29643443

[acel70224-bib-0015] Houseman, E. A. , W. P. Accomando , D. C. Koestler , et al. 2012. “DNA Methylation Arrays as Surrogate Measures of Cell Mixture Distribution.” BMC Bioinformatics 13, no. 1: 86. 10.1186/1471-2105-13-86. 22568884 PMC3532182

[acel70224-bib-0016] Hu, T. , L. Yao , K. Reynolds , et al. 2016. “The Effects of a Low‐Carbohydrate Diet on Appetite: A Randomized Controlled Trial.” Nutrition, Metabolism, and Cardiovascular Diseases 26, no. 6: 476–488. 10.1016/j.numecd.2015.11.011. PMC4873405 26803589

[acel70224-bib-0017] Hyde, P. N. , T. N. Sapper , C. D. Crabtree , et al. 2019. “Dietary Carbohydrate Restriction Improves Metabolic Syndrome Independent of Weight Loss.” JCI Insight 4, no. 12: e128308. 10.1172/jci.insight.128308. 31217353 PMC6629108

[acel70224-bib-0018] Ikram, M. A. 2024. “The Use and Misuse of ‘Biological Aging’ in Health Research.” Nature Medicine 30, no. 11: 3045. 10.1038/s41591-024-03297-9. 39375458

[acel70224-bib-0019] Joyce, B. T. , T. Gao , Y. Zheng , et al. 2021. “Epigenetic Age Acceleration Reflects Long‐Term Cardiovascular Health.” Circulation Research 129, no. 8: 770–781. 10.1161/CIRCRESAHA.121.318965. 34428927 PMC8484046

[acel70224-bib-0020] Keller, M. , A. Yaskolka Meir , S. H. Bernhart , et al. 2020. “DNA Methylation Signature in Blood Mirrors Successful Weight‐Loss During Lifestyle Interventions: The CENTRAL Trial.” Genome Medicine 12, no. 1: 97. 10.1186/s13073-020-00794-7. 33198820 PMC7670623

[acel70224-bib-0021] Leek, J. T. , W. E. Johnson , H. S. Parker , A. E. Jaffe , and J. D. Storey . 2012. “The Sva Package for Removing Batch Effects and Other Unwanted Variation in High‐Throughput Experiments.” Bioinformatics 28, no. 6: 882–883. 10.1093/bioinformatics/bts034. 22257669 PMC3307112

[acel70224-bib-0022] Levine, M. E. , A. T. Lu , A. Quach , et al. 2018. “An Epigenetic Biomarker of Aging for Lifespan and Healthspan.” Aging (Albany NY) 10, no. 4: 573–591. 10.18632/aging.101414. 29676998 PMC5940111

[acel70224-bib-0023] Lin, L. , J. Kiryakos , F. Ammous , et al. 2024. “Epigenetic Age Acceleration Is Associated With Blood Lipid Levels in a Multi‐Ancestry Sample of Older U.S. Adults.” BMC Medical Genomics 17, no. 1: 146. 10.1186/s12920-024-01914-7. 38802805 PMC11129464

[acel70224-bib-0024] Lo, Y. H. , and W. Y. Lin . 2022. “Cardiovascular Health and Four Epigenetic Clocks.” Clinical Epigenetics 14, no. 1: 73. 10.1186/s13148-022-01295-7. 35681159 PMC9185918

[acel70224-bib-0025] López‐Otín, C. , M. A. Blasco , L. Partridge , M. Serrano , and G. Kroemer . 2023. “Hallmarks of Aging: An Expanding Universe.” Cell 186, no. 2: 243–278. 10.1016/j.cell.2022.11.001. 36599349

[acel70224-bib-0026] Lu, A. T. , A. Quach , J. G. Wilson , et al. 2019. “DNA Methylation GrimAge Strongly Predicts Lifespan and Healthspan.” Aging (Albany NY) 11, no. 2: 303–327. 10.18632/aging.101684. 30669119 PMC6366976

[acel70224-bib-0027] Matthews, D. R. , J. P. Hosker , A. S. Rudenski , B. A. Naylor , D. F. Treacher , and R. C. Turner . 1985. “Homeostasis Model Assessment: Insulin Resistance and Beta‐Cell Function From Fasting Plasma Glucose and Insulin Concentrations in Man.” Diabetologia 28, no. 7: 412–419. 10.1007/bf00280883. 3899825

[acel70224-bib-0028] Mazidi, M. , N. Katsiki , D. P. Mikhailidis , N. Sattar , and M. Banach . 2019. “Lower Carbohydrate Diets and All‐Cause and Cause‐Specific Mortality: A Population‐Based Cohort Study and Pooling of Prospective Studies.” European Heart Journal 40, no. 34: 2870–2879. 10.1093/eurheartj/ehz174. 31004146

[acel70224-bib-0029] Miao, K. , X. Hong , W. Cao , et al. 2024. “Association Between Epigenetic Age and Type 2 Diabetes Mellitus or Glycemic Traits: A Longitudinal Twin Study.” Aging Cell 23, no. 7: e14175. 10.1111/acel.14175. 38660768 PMC11258448

[acel70224-bib-0030] Moqri, M. , C. Herzog , J. R. Poganik , et al. 2023. “Biomarkers of Aging for the Identification and Evaluation of Longevity Interventions.” Cell 186, no. 18: 3758–3775. 10.1016/j.cell.2023.08.003. 37657418 PMC11088934

[acel70224-bib-0031] Perri, G. , C. French , C. Agostinis‐Sobrinho , A. Anand , R. D. Antarianto , and Y. Arai . 2024. “An Expert Consensus Statement on Biomarkers of Aging for Use in Intervention Studies.” Journals of Gerontology, Series A: Biological Sciences and Medical Sciences 80, no. 5: glae297. 10.1093/gerona/glae297. PMC11979094 39708300

[acel70224-bib-0032] Salas, L. A. , D. C. Koestler , R. A. Butler , et al. 2018. “An Optimized Library for Reference‐Based Deconvolution of Whole‐Blood Biospecimens Assayed Using the Illumina HumanMethylationEPIC BeadArray.” Genome Biology 19, no. 1: 64. 10.1186/s13059-018-1448-7. 29843789 PMC5975716

[acel70224-bib-0033] Samaha, F. F. , N. Iqbal , P. Seshadri , et al. 2003. “A Low‐Carbohydrate as Compared With a Low‐Fat Diet in Severe Obesity.” New England Journal of Medicine 348, no. 21: 2074–2081. 10.1056/NEJMoa022637. 12761364

[acel70224-bib-0034] Simental‐Mendía, L. E. , M. Rodríguez‐Morán , and F. Guerrero‐Romero . 2008. “The Product of Fasting Glucose and Triglycerides as Surrogate for Identifying Insulin Resistance in Apparently Healthy Subjects.” Metabolic Syndrome and Related Disorders 6, no. 4: 299–304. 10.1089/met.2008.0034. 19067533

[acel70224-bib-0035] Tian, Y. E. , V. Cropley , A. B. Maier , N. T. Lautenschlager , M. Breakspear , and A. Zalesky . 2023. “Heterogeneous Aging Across Multiple Organ Systems and Prediction of Chronic Disease and Mortality.” Nature Medicine 29, no. 5: 1221–1231. 10.1038/s41591-023-02296-6. 37024597

[acel70224-bib-0036] Valenzuela, P. L. , P. Carrera‐Bastos , A. Castillo‐García , D. E. Lieberman , A. Santos‐Lozano , and A. Lucia . 2023. “Obesity and the Risk of Cardiometabolic Diseases.” Nature Reviews. Cardiology 20, no. 7: 475–494. 10.1038/s41569-023-00847-5. 36927772

[acel70224-bib-0037] van Iterson, M. , E. W. Tobi , R. C. Slieker , et al. 2014. “MethylAid: Visual and Interactive Quality Control of Large Illumina 450k Datasets.” Bioinformatics 30, no. 23: 3435–3437. 10.1093/bioinformatics/btu566. 25147358

[acel70224-bib-0038] Wang, Y. , O. A. Grant , X. Zhai , K. D. McDonald‐Maier , and L. C. Schalkwyk . 2024. “Insights Into Ageing Rates Comparison Across Tissues From Recalibrating Cerebellum DNA Methylation Clock.” Geroscience 46, no. 1: 39–56. 10.1007/s11357-023-00871-w. 37597113 PMC10828477

[acel70224-bib-0039] Waziry, R. , C. P. Ryan , D. L. Corcoran , et al. 2023. “Effect of Long‐Term Caloric Restriction on DNA Methylation Measures of Biological Aging in Healthy Adults From the CALERIE Trial.” Nature Aging 3, no. 3: 248–257. 10.1038/s43587-022-00357-y. 37118425 PMC10148951

[acel70224-bib-0040] Yaskolka Meir, A. , M. Keller , S. H. Bernhart , et al. 2021. “Lifestyle Weight‐Loss Intervention May Attenuate Methylation Aging: The CENTRAL MRI Randomized Controlled Trial.” Clinical Epigenetics 13, no. 1: 48. 10.1186/s13148-021-01038-0. 33663610 PMC7934393

