# Supplementary material for: Epigenetic Age Acceleration and Cardiometabolic Biomarkers in Response to Weight‐Loss Dietary Interventions Among Obese Individuals: The MACRO Trial
Source: Aging Cell. 2025 Sep 8;24(11):e70224. doi: 10.1111/acel.70224 (PMC12611269; doi:10.1111/acel.70224)
Supplement: Supplementary file 1 — Data S1: acel70224‐sup‐0001‐DataS1.docx. [file ACEL-24-e70224-s001.docx]

**Supplemental Methods**

**Trial design**

During the trial, participants were asked to maintain their physical activity levels during the intervention. A handbook containing supportive information was given to participants, including recipes, sample menus for 1 week of food intake at various energy levels, food lists, shopping lists, meal planners, and guides on counting macronutrients and reading nutrition labels. A low-carbohydrate or low-fat meal replacement (bar or shake) was also given per day to participants in each group for the duration of the study. Participants met with a dietitian in weekly individual counseling sessions for the first 4 weeks, followed by small group counseling sessions every other week for the next 5 months (a total of 10 sessions) and monthly for the last 6 months of the intervention. Staff provided a single set of instructions that were not altered over the course of the study. Participants in each diet group received the same information on dietary fiber (recommended intake of 25 g/d) and types of dietary fats. These common instructions included education on saturated, monounsaturated, and trans fats, with emphasis on the benefits of monounsaturated fats and recommendations to limit or eliminate trans fats.

**Regression model equations**

1. The associations between epigenetic age acceleration and cardiometabolic biomarkers

1) At baseline, with linear regression models

$$\boldsymbol{Log}\left( \boldsymbol{biomarkers} \right)\boldsymbol{=epigentic age acceleration+ baseline chronological age+ sex+race+ education levels+baseline smoking status+baseline alcohol use+basleine physical activity+baseline BMI+baseline total energy intake}\boldsymbol{+baseline}\boldsymbol{immune cell composition}$$

2) Post-intervention, using linear mixed-effect models

$$\boldsymbol{Log}\left( \boldsymbol{biomarkers} \right)\boldsymbol{=}\boldsymbol{corresponding}\boldsymbol{epigentic age acceleration+}\boldsymbol{corresponding}\boldsymbol{chronological age+ sex+race+ education levels+}\boldsymbol{corresponding}\boldsymbol{smoking status+}\boldsymbol{corresponding}\boldsymbol{alcohol use+}\boldsymbol{corresponding}\boldsymbol{physical activity+}\boldsymbol{corresponding}\boldsymbol{BMI+}\boldsymbol{corresponding}\boldsymbol{total energy intake+}\boldsymbol{corresponding}\boldsymbol{immune cell composition}\boldsymbol{+time+intervention groups+random intercept for each participant}$$

3) Change-to-change analysis, using linear regression models

$$\boldsymbol{Log}\left( \boldsymbol{changes in}\boldsymbol{biomarkers} \right)\boldsymbol{=}\boldsymbol{corresponding}\boldsymbol{changes in}\boldsymbol{epigentic age acceleration+}\boldsymbol{baseline}\boldsymbol{chronological age+ sex+race+ education levels+}\boldsymbol{baseline}\boldsymbol{smoking status+}\boldsymbol{baseline}\boldsymbol{alcohol use+}\boldsymbol{baseline} \boldsymbol{physical activity+}\boldsymbol{baseline}\boldsymbol{BMI+}\boldsymbol{baseline} \boldsymbol{total energy intake+}\boldsymbol{baseline}\boldsymbol{immune cell composition}\boldsymbol{+intervention groups}$$

2. The mediation analysis to test whether weight loss-induced changes in cardiometabolic biomarkers could be attributed to changes in epigenetic age acceleration over 12-month follow-up

1) The mediator model

$$\boldsymbol{Log}\left( \boldsymbol{changes in epigenetic age acceleration} \right)\boldsymbol{=}\boldsymbol{corresponding changes in BMI}\boldsymbol{+}\boldsymbol{baseline epigenetic age acceleration}\boldsymbol{+baseline BMI}\boldsymbol{+}\boldsymbol{baseline chronological age+ sex+race+ education levels+baseline smoking status+baseline alcohol use+basleine physical activity+baseline total energy intake+baseline immune cell composition}$$

2) The outcome model

$$\boldsymbol{Log}\left( \boldsymbol{changes in}\boldsymbol{biomarkers} \right)\boldsymbol{=}\boldsymbol{corresponding changes in BMI}\boldsymbol{+}\boldsymbol{corresponding changes in epigenetic age acceleration+}\boldsymbol{baseline biomarkers+}\boldsymbol{baseline epigenetic age acceleration}\boldsymbol{+baseline BMI}\boldsymbol{+}\boldsymbol{baseline chronological age+ sex+race+ education levels+baseline smoking status+baseline alcohol use+basleine physical activity+baseline total energy intake+baseline immune cell composition}$$

**Supplemental Tables**

| **Supplemental Table 1. Measures of adherence by time and dietary intervention groups** | | | | | | |
| --- | --- | --- | --- | --- | --- | --- |
| **Measures** | **Low-fat diets** | | | **Low-carbohydrate diets** | | |
|  | **Baseline** | **Three months** | **Twelve months** | **Baseline** | **Three months** | **Twelve months** |
| Dietary intake |  |  |  |  |  |  |
| Total fat, g | 80.38 (32.67) | 44.75 (21.58) | 52.06 (24.47) | 75.47 (36.92) | 63.96 (29.87) | 69.15 (35.91) |
| Total saturated fat, g | 27.37 (13.62) | 13.17 (6.70) | 15.41 (8.56) | 24.67 (14.63) | 20.28 (10.58) | 23.00 (14.76) |
| Total carbohydrates, g | 242.74 (101.27) | 194.07 (75.46) | 200.83 (76.34) | 241.52 (93.27) | 98.70 (45.17) | 128.52 (67.22) |
| Digestible carbohydrates | 226.15 (98.75) | 176.83 (69.64) | 183.90 (72.17) | 223.12 (89.31) | 82.52 (43.31) | 113.73 (62.95) |
| Proportion of energy |  |  |  |  |  |  |
| From fat, % | 34.61 (6.60) | 27.35 (8.59) | 29.21 (8.32) | 32.45 (7.28) | 42.78 (10.00) | 40.63 (10.30) |
| From saturated fat, % | 11.51 (2.90) | 7.98 (2.92) | 8.71 (3.27) | 10.47 (3.45) | 13.54 (4.31) | 13.24 (4.78) |
| From carbohydrates, % | 46.15 (7.80) | 53.07 (10.51) | 50.92 (9.35) | 48.08 (8.95) | 29.07 (12.61) | 34.40 (13.64) |
| Values are means (SD). | | | | | | |

| **Supplemental Table 2. Correlation between chronological age, epigenetic age, and epigenetic age acceleration at each visit** | | | | | | |
| --- | --- | --- | --- | --- | --- | --- |
|  | Chronological age | PCPhenoAge | PCGrimAge | DunedinPACE | PCPhenoAge Acceleration | PCGrimAge acceleration |
| Pre-intervention baseline | | | | | | |
| Chronological age | 1 |  |  |  |  |  |
| PCPhenoAge | 0.79 | 1 |  |  |  |  |
| PCGrimAge | 0.96 | 0.85 | 1 |  |  |  |
| DunedinPACE | 0.02 | 0.29 | 0.16 | 1 |  |  |
| PCPhenoAge Acceleration | <0.001 | 0.27 | 0.07 | 0.15 | 1 |  |
| PCGrimAge acceleration | <0.001 | 0.12 | 0.07 | 0.15 | 0.56 | 1 |
| Post-intervention 3-month follow-up | | | | | | |
| Chronological age | 1 |  |  |  |  |  |
| PCPhenoAge | 0.78 | 1 |  |  |  |  |
| PCGrimAge | 0.96 | 0.84 | 1 |  |  |  |
| DunedinPACE | 0.07 | 0.28 | 0.21 | 1 |  |  |
| PCPhenoAge Acceleration | <0.001 | 0.3 | 0.07 | 0.19 | 1 |  |
| PCGrimAge acceleration | <0.001 | 0.14 | 0.09 | 0.25 | 0.63 | 1 |
| Post-intervention 12-month follow-up | | | | | | |
| Chronological age | 1 |  |  |  |  |  |
| PCPhenoAge | 0.79 | 1 |  |  |  |  |
| PCGrimAge | 0.96 | 0.84 | 1 |  |  |  |
| DunedinPACE | <0.001 | 0.22 | 0.15 | 1 |  |  |
| PCPhenoAge Acceleration | <0.001 | 0.18 | 0.03 | 0.12 | 1 |  |
| PCGrimAge acceleration | <0.001 | 0.16 | 0.12 | 0.29 | 0.64 | 1 |

| **Supplemental Table 3. The associations between epigenetic age acceleration metrics and cardiometabolic biomarkers by follow-up time** | | | | | | |
| --- | --- | --- | --- | --- | --- | --- |
| **Epigenetic age acceleration  metrics** | **Cardiometabolic biomarkers** | **Three months^a^** | | **Twelve months^a^** | | **FDR for  interaction** |
|  |  | **Estimates  (95% CI)** | **FDR** | **Estimates  (95% CI)** | **FDR** |  |
| PCPhenoAge  acceleration | Glucose | -0.7 (-3-1.7) | 0.781 | 2.2 (-0.2-4.7) | 0.562 | 0.957 |
|  | Insulin | -6.7 (-17.5-5.6) | 0.584 | 4.9 (-7.3-18.7) | 0.701 | 0.957 |
|  | HOMA-IR | -7.5 (-19.2-5.9) | 0.581 | 7.3 (-6.3-22.8) | 0.701 | 0.957 |
|  | TyG index | -0.1 (-1.3-1.1) | 0.95 | -0.5 (-1.7-0.7) | 0.701 | 0.957 |
|  | Total cholesterol | -0.7 (-4-2.7) | 0.872 | -1 (-4.2-2.4) | 0.775 | 0.961 |
|  | Triglycerides | -0.2 (-8.6-9) | 1 | -6.1 (-14.2-2.8) | 0.633 | 0.957 |
|  | LDL-C | -1.1 (-6.2-4.3) | 0.872 | -0.3 (-5.4-5.1) | 0.937 | 0.957 |
|  | HDL-C | 0 (-5-5.2) | 1 | 1.2 (-3.8-6.5) | 0.775 | 0.957 |
|  | CRP | -8.1 (-24.3-11.6) | 0.713 | 8.9 (-10.2-31.9) | 0.701 | 0.957 |
|  | Adiponectin | 0 (-7.4-7.9) | 1 | -3.7 (-10.8-3.9) | 0.701 | 0.957 |
|  | Ghrelin | -4 (-9.5-1.8) | 0.428 | 0.1 (-5.6-6.2) | 0.968 | 0.957 |
|  | Peptide YY | 0.2 (-7.2-8.2) | 1 | 6.9 (-0.9-15.4) | 0.562 | 0.957 |
| PCGrimAge  acceleration | Glucose | -0.4 (-2.5-1.8) | 0.872 | 1.3 (-0.9-3.5) | 0.633 | 0.957 |
|  | Insulin | -8.1 (-17.8-2.8) | 0.393 | 1.3 (-10.1-14.2) | 0.877 | 0.957 |
|  | HOMA-IR | -8.6 (-19.2-3.4) | 0.393 | 2.6 (-10-17) | 0.785 | 0.957 |
|  | TyG index | -0.8 (-1.8-0.2) | 0.378 | -0.2 (-1.4-1) | 0.775 | 0.957 |
|  | Total cholesterol | -1.2 (-4.2-2) | 0.751 | -0.9 (-4.1-2.5) | 0.775 | 0.961 |
|  | Triglycerides | -6.3 (-13.5-1.5) | 0.378 | -3.3 (-11.3-5.4) | 0.701 | 0.957 |
|  | LDL-C | -0.8 (-5.4-4) | 0.891 | -1.7 (-6.6-3.5) | 0.758 | 0.957 |
|  | HDL-C | 0.9 (-3.7-5.8) | 0.872 | 1.9 (-3-7) | 0.701 | 0.957 |
|  | CRP | -6.1 (-21.3-12) | 0.758 | -5.9 (-22.1-13.6) | 0.758 | 0.985 |
|  | Adiponectin | -0.9 (-7.5-6.1) | 0.921 | -1.3 (-8.4-6.3) | 0.79 | 0.968 |
|  | Ghrelin | -5.8 (-10.7--0.7) | 0.12 | 1.3 (-4.5-7.4) | 0.775 | 0.957 |
|  | Peptide YY | 2 (-4.9-9.5) | 0.837 | 3.4 (-4.3-11.6) | 0.701 | 0.957 |
| DunedinPACE^b^ | Glucose | 1 (-1-3) | 0.585 | 0.5 (-1.6-2.7) | 0.775 | 0.957 |
|  | Insulin | 13.8 (1.9-27) | 0.118 | 10.7 (-1.7-24.8) | 0.562 | 0.957 |
|  | HOMA-IR | 14.7 (1.8-29.2) | 0.118 | 11 (-2.7-26.5) | 0.562 | 0.957 |
|  | TyG index | 0.5 (-0.5-1.5) | 0.585 | 0.7 (-0.5-1.9) | 0.633 | 0.957 |
|  | Total cholesterol | -3.4 (-6.4--0.4) | 0.118 | -2.7 (-5.9-0.6) | 0.562 | 0.957 |
|  | Triglycerides | 2.9 (-4.6-11.1) | 0.758 | 5.2 (-3.3-14.5) | 0.633 | 0.957 |
|  | LDL-C | -5.4 (-9.8--0.9) | 0.118 | -4.4 (-9.3-0.8) | 0.562 | 0.957 |
|  | HDL-C | -3.8 (-7.9-0.4) | 0.294 | -2.9 (-7.5-2) | 0.633 | 0.957 |
|  | CRP | 41.2 (20.2-65.8) | 0.001 | 37.3 (15.1-63.8) | 0.018 | 0.957 |
|  | Adiponectin | -12.5 (-18.8--5.8) | 0.01 | -12.1 (-19--4.6) | 0.046 | 0.957 |
|  | Ghrelin | -7.9 (-12.6--2.9) | 0.029 | -2.6 (-8-3.1) | 0.701 | 0.451 |
|  | Peptide YY | 8.9 (2.7-15.5) | 0.044 | 4.5 (-2-11.5) | 0.633 | 0.957 |
| Abbreviation: CRP, C-reactive protein; HDL, high-density lipoprotein; HOMA-IR, homeostatic model assessment for insulin resistance; LDL, low-density lipoprotein; | | | | | | |
| a. Linear mixed-effects models adjusted for chronological age, sex, race, education, smoking, alcohol use, concurrent body mass index, total energy intake, physical activity, immune cell composition, intervention groups, and the production term with follow-up time | | | | | | |
| b. Per 0.1 unit increment | | | | | | |

| **Supplemental Table 4. The associations between epigenetic age acceleration metrics and cardiometabolic biomarkers by intervention groups at the post-intervention period** | | | | | | |
| --- | --- | --- | --- | --- | --- | --- |
| **Epigenetic age acceleration  metrics** | **Measures of cardiometabolic health** | **Low-fat diets^a^** | | **Low-carbohydrate diets^a^** | | **FDR for  interaction** |
|  |  | **Estimates  (95% CI)** | **FDR** | **Estimates  (95% CI)** | **FDR** |  |
| PCPhenoAge  acceleration | Glucose | 0.4 (-1.7-2.6) | 0.863 | 1 (-1-3) | 0.539 | 0.923 |
|  | Insulin | -3.2 (-13.6-8.4) | 0.82 | 0.6 (-9-11.2) | 0.969 | 0.907 |
|  | HOMA-IR | -3 (-14.2-9.8) | 0.847 | 1.4 (-9.1-13.2) | 0.927 | 0.907 |
|  | TyG index | 0.3 (-0.7-1.3) | 0.82 | -0.8 (-1.8-0.2) | 0.363 | 0.506 |
|  | Total cholesterol | -2.3 (-5.3-0.8) | 0.478 | 0.2 (-2.5-3) | 0.969 | 0.612 |
|  | Triglycerides | 2.5 (-5.6-11.3) | 0.82 | -7.2 (-13.7--0.2) | 0.251 | 0.506 |
|  | LDL-C | -3.1 (-7.5-1.6) | 0.518 | 1 (-3.3-5.5) | 0.792 | 0.552 |
|  | HDL-C | -2.3 (-6.8-2.4) | 0.75 | 2.6 (-1.5-6.9) | 0.482 | 0.506 |
|  | CRP | -0.1 (-16.9-20.1) | 0.988 | 0.3 (-14.4-17.6) | 0.969 | 0.991 |
|  | Adiponectin | -1.1 (-7.5-5.7) | 0.863 | -2.5 (-8.2-3.6) | 0.646 | 0.923 |
|  | Ghrelin | -1.9 (-6.9-3.5) | 0.82 | -2 (-6.5-2.7) | 0.646 | 0.991 |
|  | Peptide YY | 6.7 (-1.3-15.4) | 0.478 | 1.4 (-5.1-8.4) | 0.83 | 0.667 |
| PCGrimAge  acceleration | Glucose | -0.6 (-2.5-1.4) | 0.82 | 1.3 (-0.5-3.1) | 0.42 | 0.552 |
|  | Insulin | -2.1 (-11.6-8.4) | 0.863 | -5.3 (-13.9-4.3) | 0.497 | 0.923 |
|  | HOMA-IR | -2.7 (-13.1-9.1) | 0.847 | -4.1 (-13.7-6.6) | 0.647 | 0.963 |
|  | TyG index | -0.1 (-1.1-0.9) | 0.905 | -1 (-2-0) | 0.251 | 0.612 |
|  | Total cholesterol | -1.3 (-4-1.5) | 0.763 | -0.9 (-3.4-1.7) | 0.742 | 0.963 |
|  | Triglycerides | -0.2 (-7.4-7.5) | 0.988 | -9 (-15.2--2.3) | 0.111 | 0.506 |
|  | LDL-C | -1.4 (-5.6-3) | 0.82 | -1.1 (-5.1-3.1) | 0.792 | 0.991 |
|  | HDL-C | -1.7 (-5.8-2.6) | 0.82 | 4.2 (0.2-8.4) | 0.251 | 0.506 |
|  | CRP | -12.5 (-25.9-3.4) | 0.478 | -0.4 (-14.7-16.3) | 0.969 | 0.612 |
|  | Adiponectin | -0.4 (-6.1-5.6) | 0.959 | -1.8 (-7-3.8) | 0.742 | 0.923 |
|  | Ghrelin | -2.6 (-7.2-2.3) | 0.702 | -2.6 (-6.9-1.9) | 0.497 | 0.991 |
|  | Peptide YY | 1.2 (-5.7-8.6) | 0.863 | 4 (-2.9-11.4) | 0.497 | 0.907 |
| DunedinPACE^b^ | Glucose | -0.4 (-2.7-2) | 0.863 | 2 (-0.4-4.4) | 0.31 | 0.552 |
|  | Insulin | 11.7 (-2.6-28.2) | 0.478 | 13.5 (-0.6-29.7) | 0.251 | 0.963 |
|  | HOMA-IR | 11.3 (-4.3-29.4) | 0.478 | 15.5 (-0.3-33.8) | 0.251 | 0.923 |
|  | TyG index | 0.8 (-0.4-2) | 0.478 | 0.3 (-0.9-1.5) | 0.792 | 0.907 |
|  | Total cholesterol | -2.8 (-6.5-1.1) | 0.478 | -3.5 (-7.1-0.1) | 0.251 | 0.953 |
|  | Triglycerides | 7.5 (-2.2-18.1) | 0.478 | -0.2 (-9-9.4) | 0.969 | 0.612 |
|  | LDL-C | -6.4 (-11.9--0.5) | 0.366 | -3.8 (-9.3-2) | 0.474 | 0.907 |
|  | HDL-C | -2.4 (-7.6-3.1) | 0.763 | -4.5 (-9.4-0.7) | 0.31 | 0.907 |
|  | CRP | 23.2 (1.1-50.2) | 0.366 | 57.1 (29.4-90.8) | <0.001 | 0.506 |
|  | Adiponectin | -9.4 (-18-0.1) | 0.388 | -14.7 (-22.2--6.5) | 0.014 | 0.767 |
|  | Ghrelin | -8.5 (-14.4--2.2) | 0.228 | -3.7 (-9.8-2.7) | 0.497 | 0.612 |
|  | Peptide YY | 8.8 (2-16) | 0.228 | 5.1 (-1.7-12.4) | 0.402 | 0.907 |
| Abbreviation: CRP, C-reactive protein; HDL, high-density lipoprotein; HOMA-IR, homeostatic model assessment for insulin resistance; LDL, low-density lipoprotein; | | | | | | |
| a. Linear mixed-effects models adjusted for chronological age, sex, race, education, smoking, alcohol use, concurrent body mass index, total energy intake, physical activity, immune cell composition, time, and the production term with intervention groups | | | | | | |
| b. Per 0.1 unit increment | | | | | | |

**Supplemental Figures**


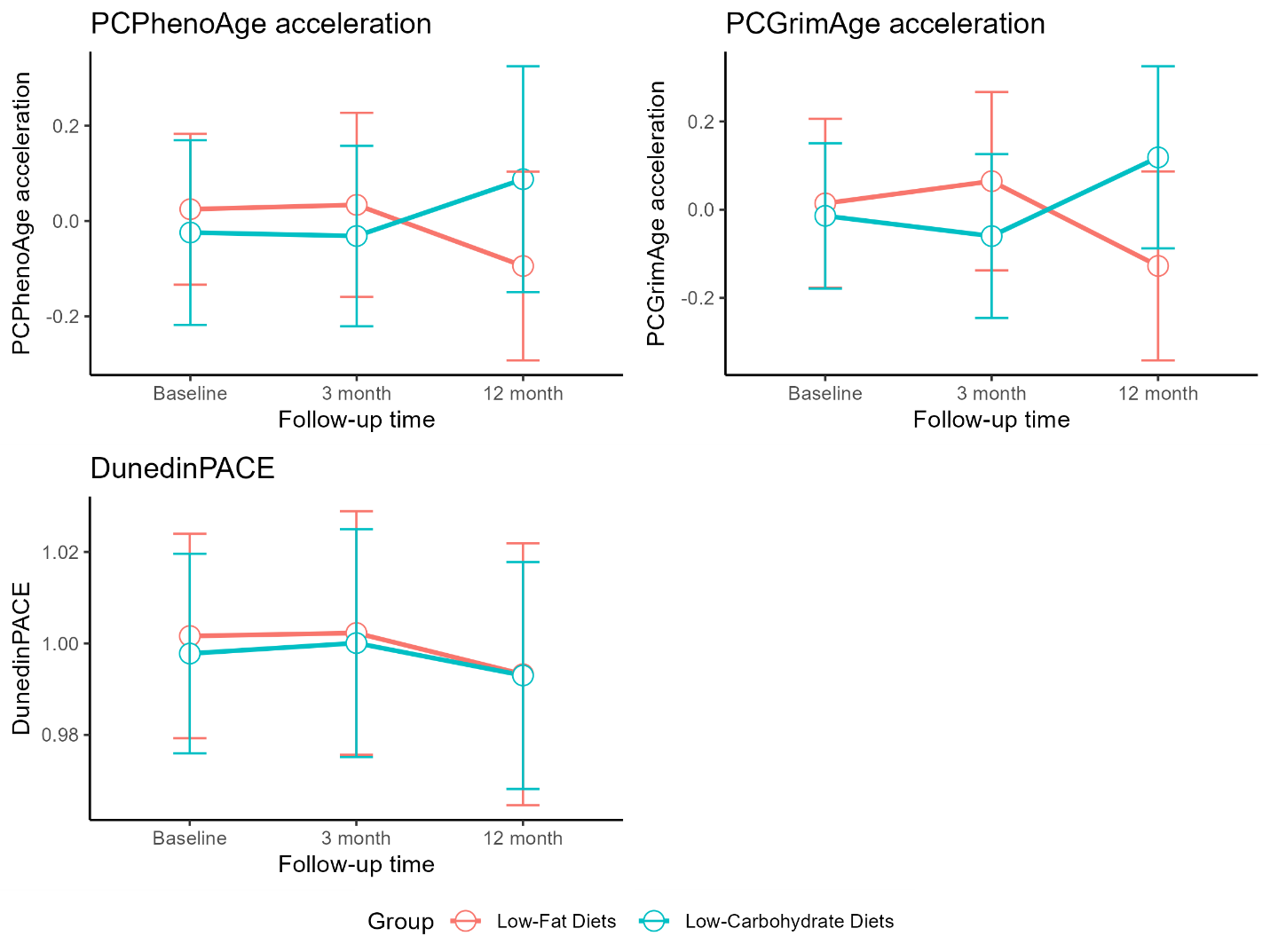


Supplemental Figure 1. The trajectories of epigenetic age acceleration metrics by intervention groups. (A) PCPhenoAge acceleration. (B) PCGrimAge acceleration. (C) DunedinPACE. Circles represent the average values of epigenetic age acceleration metrics. Bars represent the 95% confidence intervals of the average values.
